# Microfluidic-Based Mechanical Phenotyping of Androgen-Sensitive and Non-sensitive Prostate Cancer Cells Lines

**DOI:** 10.3390/mi10090602

**Published:** 2019-09-12

**Authors:** Na Liu, Panpan Du, Xiaoxiao Xiao, Yuanyuan Liu, Yan Peng, Chen Yang, Tao Yue

**Affiliations:** 1 School of Mechatronics Engineering and Automation, Shanghai University, Shanghai 200444, China; liuna_sia@shu.edu.cn (N.L.); dpp_shu@163.com (P.D.); xxx229471513@163.com (X.X.); yuanyuan_liu@shu.edu.cn (Y.L.); 2 Fudan Institute of Urology, Fudan University, Shanghai 200433, China

**Keywords:** cellular mechanics, cell deformability, single-cell analysis, high-throughput, microfluidic, morphological rheology

## Abstract

Cell mechanical properties have been identified to characterize cells pathologic states. Here, we report our work on high-throughput mechanical phenotyping of androgen-sensitive and non-sensitive human prostate cancer cell lines based on a morphological rheological microfluidic method. The theory for extracting cells’ elastic modulus from their deformation and area, and the used experimental parameters were analyzed. The mechanical properties of three types of prostate cancer cells lines with different sensitivity to androgen including LNCaP, DU145, and PC3 were quantified. The result shows that LNCaP cell was the softest, DU145 was the second softest, and PC3 was the stiffest. Furthermore, atomic force microscopy (AFM) was used to verify the effectiveness of this high-throughput morphological rheological method.

## 1. Introduction

Prostate cancer is a commonly diagnosed cancer and the second leading cause of cancer-related death in men [1,2,3]. The growth of prostate cancer is initially androgen-dependent, so androgen-deprivation therapy (ADT) has been a standard-of-care, first-line therapy for androgen-responsive prostate cancer. Unfortunately, the majority of prostate cancer patients eventually relapse into an androgen-independent growth stage, which becomes refractory to hormonal therapy and is associated with poor prognosis [4,5]. Clinically, an androgen-independent stage is diagnosed by monitoring the response of prostate specific antigen (PSA) concentration to the ADT for several months [6]. The early identification and prediction of androgen-independent prostate cancer still represent a major clinical challenge and the underlying mechanisms of the progression are not fully understood [7,8].

There has been increasing evidence that mechanical properties of cells can be used as label-free markers to determine cell physiology or pathology, such as cell metastatic potential, and differentiation degree [9,10,11,12,13]. For example, cancer cells possess lower elastic modulus than normal cells [12,14]; the stiffness of cancer cells is closely related to metastatic potential [15]; the stiffness of red blood cells in cytoskeletal disorders (malaria and sickle cell anemia) will change [16,17,18,19,20]; the stem cell deformability will change during its differentiation process [21]. So far, a variety of techniques have been developed to quantify the mechanical properties of cells, including atomic force microscopy (AFM) [22,23], magnetic twisting cytometry [24], micropipette aspiration [25,26], and optical stretching [27]. Although those biophysical approaches successfully assess cell mechanical properties, they possess drawbacks of low-throughput analysis, labor intensity or expensive equipment. In order to overcome those shortcomings, kinds of microfluidic chips have been developed for characterizing the cell mechanical properties leveraging fluid flow or geometric constrictions to deform cells [11,12,28,29,30,31]. Among them, a morphological rheological microfluidic chip [30], in which the spherical cells can deform into bullet-like shape under the fluidic shear stress when they flow through the microchannel, has attracted wide attention recently. Compared with other techniques such as AFM, micropipette aspiration, etc., this morphological rheological method could characterize the stiffness of single cells in a high-throughput way and simple structure. Different from the hydrodynamic stretching method based on cross geometry [11], Otto et al. [30] simplify the channel geometry and reduce the frame rate of high-speed camera shooting (1000–2000 f.p.s.), which seems favorable. However, previously this morpho-rheological related research all mostly measured the deformability of HL-60 cells or whole blood cells [16,21,30], and it is not clear whether epithelial tumor cells can be deformed observably due to flow through this bottlenecked microfluidic channel. In this work, we built on their approach but optimized the microfluidic chip to deform tumor cells, and develop a cell contour extraction method based on our platform.

In order to reveal the underlying difference in mechanical properties between androgen-sensitive and androgen-non-sensitive prostate cancer cells lines, this morphological rheological method was utilized to quantify the mechanical properties of three types of prostate cancer cells lines (PC3, DU145, and LNCaP) at a high-throughput of over 100 cells/s. The results show that androgen-non-sensitive prostate cancer cells line PC3 and DU145 are stiffer than androgen-sensitive prostate cancer cells line LNCaP, which suggests that the mechanical properties of prostate cancer cells can be used as potential biomarkers for early identification of androgen-independent prostate cancer.

## 2. Materials and Methods 

### 2.1. Device Setup and Working Principle

**Figure 1:**
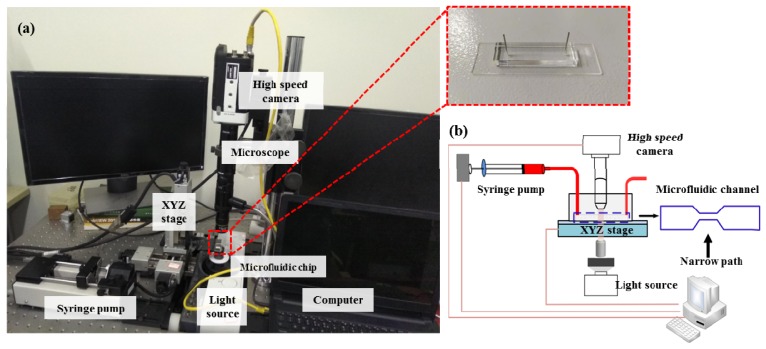
(**a**) The overview of the experimental system device (inset shows polydimethylsiloxane (PDMS) microfluidic chip), (**b**) its detailed work schematic diagram and microfluidic channel geometry.

Figure 1 provides an overview of the setup. The whole experimental system consisted of a microfluidic chip, a high-speed camera (Phantom MIRO R311, Vision Research, New Jersey, NJ, USA) integrated with a microscope (Navitar, Rochester, NY, USA), a high-power LED light, and a syringe pump (longer, Longer Precision Pump, Hebei, China). To quantify the cells’ mechanical properties, the prepared cells were pumped into the microfluidic chip using the syringe pump at an injection rate of 0.2 μL/s. The injected cells flowed through the microfluidic channel and deformation was induced by the flow shear stress and pressure gradient (Video S1). The deforming process of the cell was captured using the high-speed camera at a frame rate of 3200 frames per second and an exposure time of 5 μs. The cell deformation contour was extracted using an image processing algorithm. Compared with the previously reported chip and protocol in References [21,30], the experimental parameters including microchannel size, flow rate, flow viscosity, and frame rate were all optimized for characterizing the mechanical properties of prostate cancer cell lines due to their greater stiffness than blood cells.

A silicon-based mask manufactured using a traditional lithography process was utilized to fabricate the polydimethylsiloxane (PDMS)-based chip. The PDMS layer was manufactured as follows: the prepolymer mixed with the curing agents (Sylgard 184, Dow Corning, Midland, MI, USA) in a ratio of 10:1 was poured into the prefabricated mask and cured for 30 min at 80 °C. Then the cured PDMS film was carefully peeled off and bonded with a glass substrate through an oxygen plasma process (Diener electronic, Ebhausen, Germany). In this study, the constriction channel on the microfluidic chip possessed a length of 300 μm and a cross-section of 25 μm × 25 μm. The size of the channel was larger than the measured cells which have an averaged diameter of ~20 μm, which enabled the cell to be deformed by flow shear stress, not by the channel walls.

In this work, AFM was used to verify the accuracy of this morphological rheological method. The AFM experiment was performed with the Bioscope Resolve AFM (Bruker, Santa Barbara, CA, USA) which was integrated with an inverted microscope (Ti, Nikon, Tokyo, Japan). The PFQNM-LC-A-CAL probes (Bruker AFM Probes, Santa Barbara, CA, USA) with spring constants of 0.1 N/m, a tip radius of 65 nm, and a tip half-angle of 18° were used for the experiment. In this experimental case, a linearized Hertz model equation can be used to fit the experimental data into the spherical indenters to calculate Young’s modulus of the cell [32]:

(1) $$F^{2/3}={\left(\frac{4}{3}\frac{E}{\left(1-\mu^{2}\right)}\sqrt{R}\right)}^{2/3}\delta ,$$

where *F* is the applied loading force of the AFM probe, *E* is the Young’s modulus of the cell, *δ* is the indentation depth, *μ* is the Poisson’s ratio, and *R* is the contact radius. Cells are generally considered to be incompressible materials, so we used a Poisson’s ratio value of 0.5 [33].

### 2.2. Sample Preparation

All prostate cancer cells lines used in this report were purchased from ScienCell (Zhongqiaoxinzhou Biotech, Shanghai, China). DU145 and LNCaP cells were cultured in RPMI 1640 medium in a 5% CO_2_ humidified atmosphere at 37 °C. PC3 cells were cultured in F12 medium supplemented with 10% fetal bovine serum (HyClone, Chicago, IL, USA) and 1% penicillin/streptomycin (HyClone, Chicago, IL, USA) in a 5% CO_2_ humidified atmosphere at 37 °C. To prepare the cell suspension, cells in a petri dish were firstly washed twice using phosphate buffer saline (PBS) (HyClone, Chicago, IL, USA) to remove dead cells and other impurities. Then the cells were trypsinized from the petri dish using 0.25% Trypsin/EDTA (HyClone, Chicago, IL, USA), followed by centrifugation at 1000 rpm. Then, the cells were suspended again using PBS buffer which contained methylcellulose (Sigma-Aldrich, Saint Louis, MO, USA) at a concentration of 0.5% (w/v). Here, the methylcellulose was used to reduce cell sedimentation and increase the shear viscosity [30]. The cell concentration was kept about at 10^5^–10^6^ cells/mL. The prepared cells were transferred to a syringe for further experiments.

In the AFM experiments, all measurements were completed within two hours to ensure that the cells were in physiological state and adherent during the imaging process. 

### 2.3. Image Processing and Data Analysis

**Figure 2:**
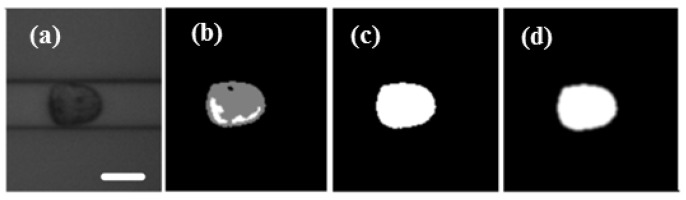
(**a**) The region of interest of video was determined and the image smoothed with Gaussian filtering. (**b**) Background subtraction. (**c**) The hole in the contour was filled. (**d**) The image was filtered and the contour was processed by convex hull. Scale bar: 20 μm.

A self-developed cell contour extraction method was modified on the real-time deformability cytometry (RT-DC) method [30]. The image processing algorithm was written by C++ code combined with OpenCV computer version library. The extraction process is shown in Figure 2. Firstly, the region of interest (ROI) was determined. Then the image was smoothed by Gaussian filtering, followed by subtracting background, filling the hole in the contour and the convex hull process. Here, the convex hull area and convex hull perimeter were used to calculate the deformation degree of cells according to Equation (2).

(2) $$D=1-\frac{4\pi A}{L^{2}},$$

where, *A* and *L* are the area and perimeter of the extracted cell, respectively. *D* is defined as the deformation of the cell.

The extracted contour data of cells was then analyzed by MATLAB R2014b and GraphPad Prism7.

### 2.4. Iso-Elasticity Lines Model

**Figure 3:**
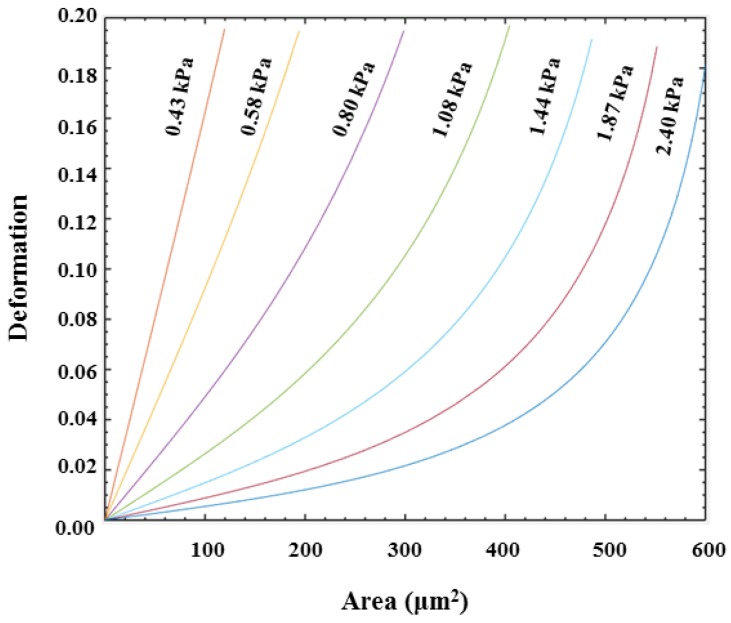
Iso-elasticity lines for elastic moduli as a function of deformation and cell size.

In the microfluidic channel, cell deformation depends not only on its elastic modulus, but also on its size, the channel size, and flow rate [30]. Reports [34,35] try to give the relationship between cell size, deformation and cell’s elastic modulus by taking advantage of an analytical expansion of the Stokes equation [34]. For different flow rates and channels, the coupling relationship between size, deformation, and stiffness is just proportional to scale. The equation can be described as

(3) $$E_{0}^{\prime }=\frac{Q^{\prime }\eta^{\prime }L^{3}}{Q{\eta L}^{\prime 3}}*E_{0},$$

where, *Q* represents the flow rate, *η* is the viscosity, *L* is channel size, and *E*_0_ is the elastic moduli by simulation [34]. *Q*’, *η*’, *L*’ represent the corresponding experimental parameters, respectively. In this work, the injection rate *Q*’ was 0.20 μL/s. According to this equation, the iso-elasticity lines were simulated as shown in Figure 3.

## 3. Results and Discussion

### 3.1. Velocity Distribution and Determined ROI

**Figure 4:**
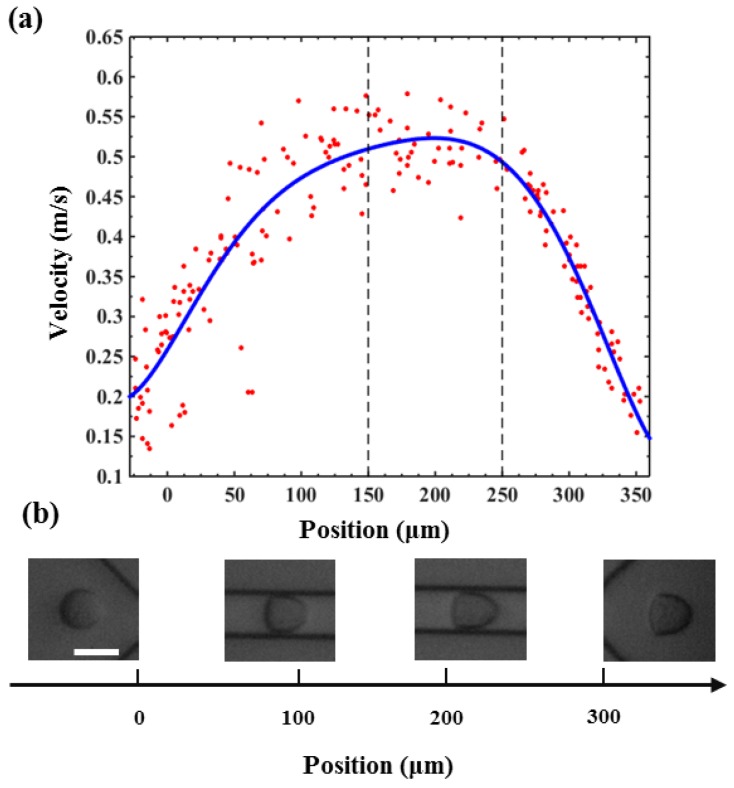
(**a**) The velocity distribution of a PC3 cell when flowing through the microfluidic constriction channel. (**b**) The morpho-rheological snapshots of a PC3 cell in the microfluidic constriction channel. Scale bar: 25 μm.

**Figure 5:**
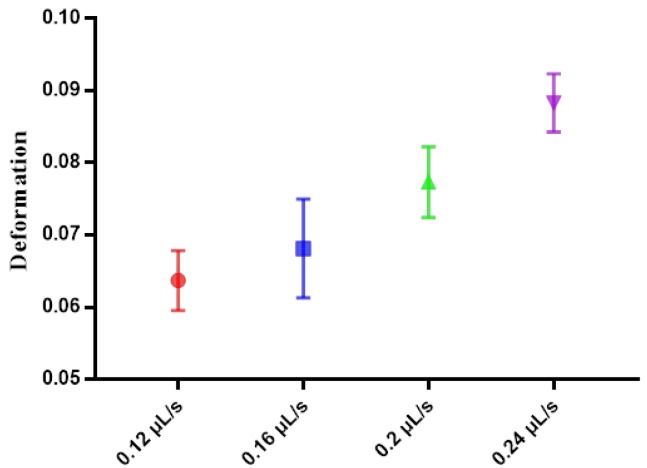
The deformation of PC3 cells using different flow rates.

In this section, the velocity distribution of PC3 cell in the 300 μm microfluidic constriction channel with an injection rate of 0.20 μL/s was analyzed. As shown in Figure 4a, the velocity of the cell would sharply increase from 0.2 m/s to a velocity of ~0.45 m/s once they had flowed into the channel at a distance of ~100 μm. Then the velocity would slightly increase to a maximum value after flowing a distance of ~200 μm, following a decreasing flowing velocity until they flowed out of the constriction channel. The cells had the highest velocity during flowing in the fragment from 150 μm to 250 μm, indicating the fluid shear stress and pressure gradients exerted on the cell reached a maximum value. In order to display the flow velocity distribution at different locations and the forces acting on the cells more intuitively, we show the morpho-rheological of the cells at different locations. It is obvious that the cell morphology changes were consistent with the velocity distribution. At the entrance and exit of the microfluidic constriction channel, the cells were close to an ideal circle, and as they flowed into the channel, the deformation increased. The cells deformation was most pronounced from 150 μm to 250 μm. Therefore, this region was set to the region of interest (ROI) for extracting cell deformation. Because the flowing velocity of the cell in the constricted channel directly depended on the injected rate of the cell suspension solution, the inject cell suspension solution rate also directly affected the cell deformation. As shown in Figure 5, the cell deformation increased with the increase of the injected rate. What needs to be noted is that the choosen injection rate should ensure simultaneously an obvious cell deformation and enough time to be detected by the high-speed camera to reduce motion blurring.

### 3.2. Mechanical Phenotyping of Prostate Cancer Cells

**Figure 6:**
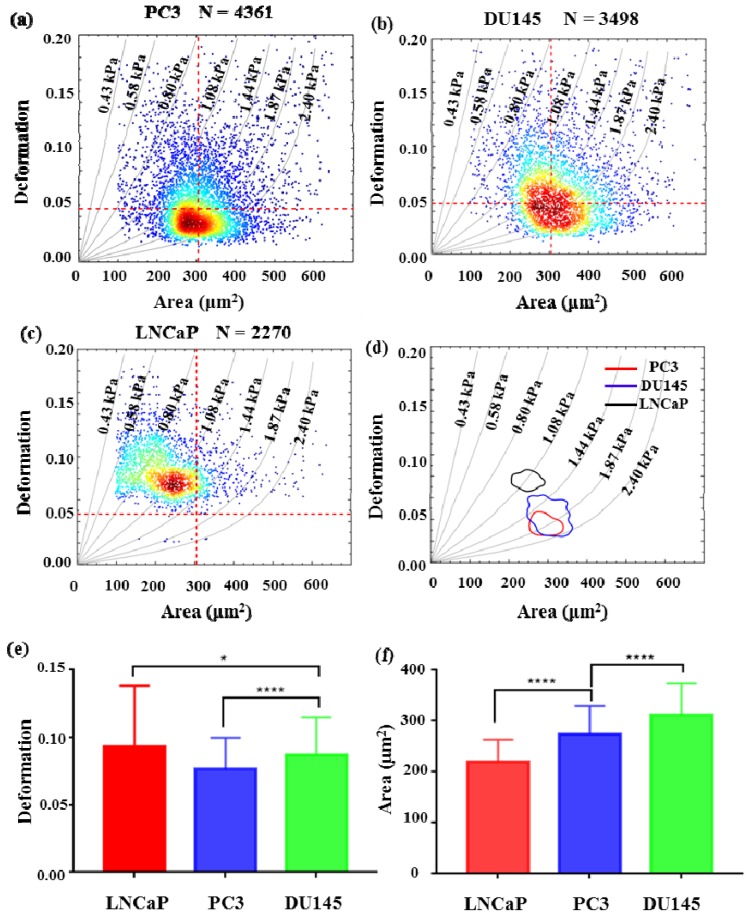
The mechanical phenotyping of prostate cancer cell lines. The scatter plot of cell deformation versus cell area (**a**) PC3, (**b**) DU145, (**c**) LNCaP. Each dot represents the information of a single cell. (**d**) The 50% density contour line of three types of prostate cancer cell lines. (**e**) The averaged deformation degree of three types of prostate cancer cell lines. (**f**) The averaged area of three types of prostate cancer cell lines. (* indicates a *p* value of less than 0.05, **** indicates a *p* value of less than 0.0001).

**Figure 7:**
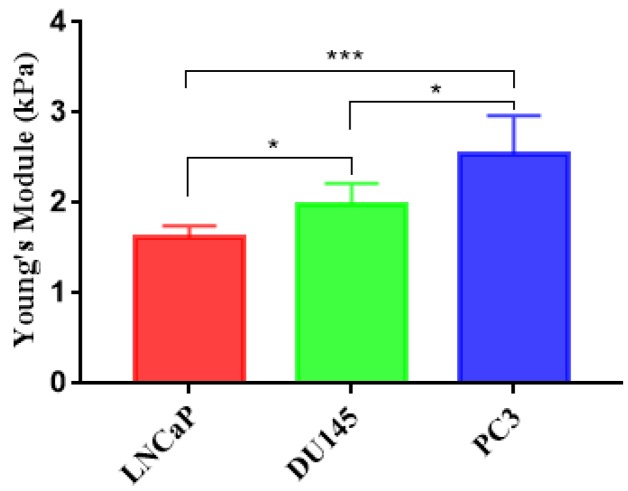
Apparent Young’s modulus of prostate cancer cells were measured using atomic force microscopy (AFM). LNCaP (1.613 ± 0.06403, n = 20), DU145 (1.984 ± 0.112, n = 25), PC3 (2.538 ± 0.2072, n = 25). (* indicates a *p* value of less than 0.05, *** indicates a *p* value of less than 0.001).

In this report, the deformation degree and elastic modulus of three types of prostate cancer cell lines PC3, DU145, and LNCaP were quantified with an injection rate of 0.2 μL/s. PC3 and DU145 are androgen-non-sensitive prostate cancer cells lines, while LNCaP is an androgen-sensitive prostate cancer cells line. Figure 6a–c are density scatter plots which show the measured cross-sectional area versus deformation degree of PC3, DU145, and LNCaP cells, respectively. In Figure 6b,c, the red dotted line for reference is the median of PC3 cell cross-sectional area and deformation. Figure 6d shows the comparison of the deformation degree and area of three types of cell lines using a density contour line which includes a 50% maximal event density. According to the simulated iso-elasticity lines in Figure 6d, the elastic modulus of three prostate cancer cell lines could be extracted, where the LNCaP cells were ~1.08 kPa, DU145 cells were 1.44–2.4 kPa, and PC3 cells were 1.87–2.40 kPa. The measured results showed the three types of prostate cancer cells possessed significant differences in their stiffness and area. LNCaP cells were softer than PC3 and DU145. Although PC3 cells had a degree of overlap with DU145, PC3 cells were slightly stiffer than DU145 cells. This trend in the stiffness of three types of cells was also verified by our AFM measured results. As shown in Figure 7, the measured apparent modulus of LNCaP, DU145, and PC3 were respectively 1.613 ± 0.06403 kPa (n = 20), 1.984 ± 0.112 kPa (n = 25), and 2.538 ± 0.2072 kPa (n = 25), which is consistent with reported results measured using AFM [14,22]. As shown in Figure 6e,f, the averaged deformation degree and area of these three prostate cancer cells lines were compared. Obviously, the size of LNCaP cells was the smallest, and the size of DU145 was the biggest. The LNCaP cells had the biggest deformation degree, while the PC3 cells had the lowest deformation degree. The results suggested that the cell size and deformation degree were also potential parameters for classifying cells type. 

Here, LNCaP, DU145, and PC3 are prostate cancer cell lines with low, moderate, and high metastatic potential, respectively [20]. According to previous reports [12,14,22], cancer cells with higher metastatic potential should possess lower stiffness than cancer cells with lower metastatic potential. However, this point is opposite to the measured results in this report. The ADT process may be responsible for these opposite results, because ADT is the dominant factor inducing androgen-sensitive prostate cancer to progress into androgen-non-sensitive prostate cancer. Previous studies have shown that androgen deprivation could lead to epithelial–mesenchymal transition (EMT) and cytoskeleton reorganization in mouse and human prostate cancer cells [36,37]. Both EMT and cytoskeleton reorganization can change cellular mechanical properties [38,39]. Additionally, ADT-caused deregulated androgen signaling is an important factor in prostate cancer progression and its pivotal role in modulating androgen-mediated EMT induction has been suggested, but the mechanistic explanations about how the interplay between ADT and EMT requires further investigation [40,41,42]. These cues suggest that the mechanical properties of prostate cancer cells may change during executing ADT, which is the possible reason that androgen-non-sensitive prostate cancer cell lines (e.g., PC3 and DU145) are stiffer than androgen-sensitive prostate cancer cell line (e.g., LNCaP). It is also suggested that cellular mechanical properties are potential biomarkers for early identification and prediction of androgen-non-sensitive prostate cancer. However, the underlying causes of this phenomenon need to be further explored in clinical samples.

## 4. Conclusions

In this work, we have applied a morphological rheological microfluidic method to quantify the mechanical properties of three types of prostate cancer cell lines (PC3, DU145, and LNCaP) at a throughput of over 100 cells/s. The velocity distribution of cells during flowing through the narrow channel, and the deformation degree of cells induced by different flow rate was analyzed. The result showed that the elastic modulus of androgen-non-sensitive prostate cancer cell lines (e.g., PC3 and DU145) were larger than that of an androgen-sensitive prostate cancer cell line (e.g., LNCaP), which was also supported by the results measured using AFM. The results suggest that the mechanical properties of prostate cancer cells can be used as potential biomarkers to identify and predicate the difference of androgen-sensitive and androgen-non-sensitive prostate cancer cell lines. This morphological rheological microfluidic method may be an alternative technique for illustrating how the androgen-sensitive prostate cancer progress into androgen-non-sensitive prostate cancer during executing ADT from a cellular mechanical view.

## Supplementary Materials

The following are available online at https://www.mdpi.com/2072-666X/10/9/602/s1, Video S1: Cells flowed through microfluidic channel and deformation was induced by the flow shear stress and pressure gradient.
